# HLA-B*51 Beyond Behcet’s Disease: Topography of Symptoms, Associated Diagnoses, and Characterization of Chronic Inflammatory Arthritis Phenotypes

**DOI:** 10.3390/ijms27093721

**Published:** 2026-04-22

**Authors:** Cinzia Rotondo, Giuseppe Busto, Raffaele Barile, Giulio Giancaspro, Brunella Capuano, Valeria Rella, Francesco Paolo Cantatore, Addolorata Corrado

**Affiliations:** Rheumatology Unit, Department of Medical and Surgical Sciences, Azienda Ospedaliero-Universitaria Policlinico Riuniti di Foggia, Università degli Studi di Foggia, 71122 Foggia, Italy

**Keywords:** spondyloarthritis, psoriatic arthritis, HLA-B*51, Behcet’s disease, chronic arthritis, rheumatoid arthritis, ankylosing spondylitis, sacroiliitis, b-DMARDs, biologic drugs, TNFα inhibitors

## Abstract

In the clinical context of the new medical concept of diseases related to the Major Histocompatibility Complex class I (MHC-I-opathy), contrasting data are available on the possible association among HLA-B*51, Behcet’s disease (BD), and spondyloarthritis (SpA). The aim of this retrospective study on a cohort of HLA-B*51-positive patients who were clinically observed for almost 5 years is primarily to evaluate which classification criteria they satisfy among BD, axial (ax) or peripheral (p) SpA, and psoriatic arthritis (PsA). Furthermore, we characterized the possible impact of different arthritis phenotypes on the most frequent extra-articular clinical manifestations in BD, ax-SpA, p-SpA, and PsA. A comparison with HLA-B*51-negative patients (matched with HLA-B*51-positive patients for age, gender, and diagnosis, by mean propensity score) was also performed to evaluate the true impact on clinical manifestations of HLA-B*51. We conducted a monocentric retrospective study from 2013 to 2025. The inclusion criteria were HLA-B*51 positivity, the availability of the entire MHC-I class test, and rheumatological clinical follow-up of at least 5 years. The exclusion criterion was positivity for clinically important MCH-I loci other than HLA-B*51. A total of 105 patients met the inclusion criteria in an average clinical observation period of 8.4 ± 2.9 years. All patients were Apulian and were HLA-B* 51 positive. During the follow-up, 32 patients (31%) met the BD criteria, 17 (16%) met the PsA criteria, 25 (24%) met the p-SpA criteria, and 13 (12%) met the ax-SpA criteria. Of note, 16% and 34% of BD patients met the ax-SpA and p-SpA ASAS criteria, respectively. Prevalent articular phenotypes in this HLA-B*51 cluster of patients are a polyarticular pattern and enthesis involvement in all disease groups. In BD patients, axial involvement was associated with a significantly higher percentage of neurological manifestations (40% vs. 7%, *p* = 0.043) and inflammatory bowel disease (IBD) (100% vs. 15%, *p* = 0.0001), compared to patients with exclusive peripheral joint involvement. This latest data on IBD remains significant, even in comparison with HLA-B*51 negative patients (33%; *p* = 0.035). In the p-SpA group, a significantly higher rate of uveitis (28%) was observed compared to both ax-SpA with HLA-B*51-positive (0%, *p* = 0.035) and p-SpA with HLA-B*51-negative patients (4%, *p* = 0.030). A high percentage of multi-drug failures was highlighted in patients with PsA (60%) and p-SpA (40%). This study provides new data on the association between HLA-B*51 and the onset of BD and/or SpA or PsA, and its possible impact on extra-articular manifestations. We confirm the higher prevalence of the peripheral articular phenotype in BD, but we also highlight a specific association between the rarer axial involvement and gastrointestinal involvement in HLA-B*51 patients. In SpA, the peripheral articular phenotype appears to be associated with a higher occurrence of uveitis in the presence of HLA-B*51.

## 1. Introduction

Growing scientific evidence tends to strengthen the new medical concept of diseases related to the Major Histocompatibility Complex class I (MHC-I-opathy) introduced in the last decade to describe a group of chronic inflammatory diseases that share a genetic, pathogenetic basis and common symptoms, such as ankylosing spondylitis (SA), psoriasis (PsO), psoriatic arthritis (PsA), acute anterior uveitis (AU), Behçet’s disease (BD), and Birdshot’s disease [1]. Indeed, there are known genome-wide association studies (GWAS) that demonstrate the relationship between BD and Human Leukocyte Antigen (HLA)-B*51 [2,3], PsO and HLA-C*06:02 [4,5,6], spondyloarthritis (SpA) and HLA-B*27 [7], AU and HLA-B*27 [8], and Birdshot uveitis and HLA-A*29 [9,10]. Old and new knowledge demonstrate that these MHC-I alleles are not just markers of pathology, but true active participants in tissue damage [11,12,13], through various mechanisms such as features in peptide presentation to CD8+ T lymphocytes, interaction with enzymes that “cut” proteins for presentation (such as ERAP1 and ERAP2), aberrant recognition of MHC-I molecules on the cell surface by Natural Killer cells T lymphocytes, and misfolding, which causes a state of cellular stress that triggers inflammation [14,15,16].

Although in many pathologies, such as SpA and Birdshot’s disease, the pathogenetic role of HLA-B*27 and HLA-A*29, respectively, seems to be well-understood [17], much remains unclear in the association between BD and HLA-B*51, which is mainly due to a different frequency of HLA-B*51 presence in BD patients in different ethnic groups. Although HLA-B*51 is known to be implicated in defective antigen presentation and cause T-cell and neutrophil dysfunction in patients with BD [18,19], these same abnormalities have been found in transgenic mice that do not develop BD [20]. Furthermore, HLA-B*51 appears to be associated with more severe disease (especially in males and for ocular involvement) [21,22]. Still, there is no clear evidence of its predictive role for serious complications or arterial aneurysms [23], except for an observed increased risk of developing genital ulcers and a lower risk of gastrointestinal manifestations [24].

Of great rheumatological interest is the debate on the presence of specific arthritis phenotypes in BD, particularly axial involvement, and the potential effect of HLA-B*51 in patients with SpA or PsA. In fact, high prevalence of musculoskeletal manifestations is known in BD, with a greater incidence of peripheral arthritis, and a variable low rate of radiographic sacroiliitis [25,26].

Recent studies on the double positivity for HLA-B*27 and B*51 (observed in 2–5% of Mediterranean cohorts) have shown a higher frequency of sacroiliitis in patients with BD [27]. In contrast, in patients with SpA, it correlates with more widespread peripheral joint involvement, mucocutaneous manifestations (particularly oral aphthae), and uveitis [28]. Few data are available on the prevalence of PsA in BD patients [29]. On the other hand, published studies suggest that, in patients with SpA, the presence of HLA-B*51 is negatively associated with sacroiliitis, it increases the risk of recurrent oral ulcerations and juxta-articular radiographic erosions [27], and it can predominantly direct the immune system towards non-bone targets [30]. So, the prospect of a unifying concept for SpA and BDs is further strengthened [30].

In this context, we present a retrospective study of a cohort of HLA-B*51-positive patients who were clinically observed for almost 5 years, primarily evaluating which classification criteria they met for BD, axial (ax) or peripheral (p) SpA, and PsA. Furthermore, we characterized the different arthritis phenotypes and the most frequent extra-articular clinical manifestations in BD, ax-SpA, p-SpA, and PsA. A comparison with HLA-B*51-negative patients (matched with HLA-B*51-positive patients for age, gender, and diagnosis) was also performed to evaluate the true impact on clinical manifestations of HLA-B*51.

## 2. Results

Of the 480 patients examined, 105 HLA-B*51-positive patients met the inclusion criteria, with an average clinical observation period of 8.4 ± 2.9 years.

In HLA-B*51-positive patients, the presenting symptoms that led patients to access the Rheumatology Unit were myalgia (47%), arthralgia (45%), fever (14%), skin lesions (12%), and ocular lesions (10%). During the follow-up, 32 patients (31%) met the BD criteria (81% International Study Group (ISG) criteria, and 84% International Criteria for Behçet’s Disease (ICBD) criteria), 17 (16%) PsA criteria, 25 (24%) p-SpA criteria, and 13 (12%) ax-SpA criteria. Only two patients developed rheumatoid arthritis characterized by bone erosion on X-rays, rheumatoid factors, and anti-citrullinated protein antibodies. The demographic and clinical characteristics of patients are shown in Table 1. Of note, 16% and 34% of BD patients met the ax-SpA and p-SpA ASAS criteria, respectively.

The “Controls”, apart from myalgia, presented a higher percentage of skin lesions, posterior uveitis, IBD, and recurrent oral aphthosis (Table 1).

Furthermore, 87 HLA-B*51 negative patients, who met the inclusion criteria, were selected by propensity score matching: 32 with BD, 13 with ax-SpA, 25 with p-SpA, and 17 with PsA. The comparison between patients who were HLA-B*51 positive and those who were HLA-B*51 negative is shown in Table 2.

All patients in this study were Apulian. At the first visit, 60% of patients declared that they used NSAIDs (in 35% of cases for more than 10 days per month), and none were using DMARDs.

### 2.1. Arthritis Phenotypes and Extra-Articular Manifestations

Prevalent articular phenotypes in this HLA-B*51 cluster of patients were a polyarticular pattern and enthesis involvement in all disease groups: BD, SpA, and PsA (Table 1).

Axial involvement was observed in 16% of BD patients and in 41% of PsA patients, as well as in patients with ax-SpA.

To better characterize the potential impact that the different phenotypes of joint involvement, axial or peripheral, can have on extra-articular manifestations, we proceeded to compare the frequency of such manifestations for each disease under study.

In BD patients, axial involvement was associated with a significantly higher percentage of neurological manifestations and IBD, compared to patients with exclusive peripheral joint involvement. The IBD frequency remained significantly higher in the group of patients with BD that were HLA-B*51 positive compared to patients with BD that were HLA-B*51 negative (Table 3).

A tendency towards a higher frequency of vascular involvement and serositis was observed in the group of patients with exclusively peripheral involvement (Table 3).

In the SpA group, the peripheral pattern was associated with a significantly higher rate of uveitis, predominantly involving the posterior segment. A significantly higher frequency of uveitis was also observed in patients with axial involvement that were HLA-B*51 positive compared to patients with axial involvement that were HLA-B*51 negative (Table 3).

Furthermore, in the peripheral arthritis pattern, there was more of a trend toward a higher rate of serositis, psoriasis, and cutaneous lesions than in the group with axial involvement (Table 3).

No significant differences were found in PsA patients between peripheral and axial involvement. Notably, a trend to an elevated rate of recurrent oral ulcerations and uveitis was observed in the peripheral arthritis group (Table 3).

### 2.2. Treatment Response

All patients were treated with cs-DMARDs except those with ax-SpA. The rates of patients treated with b-DMARDs were reported in Table 3.

In particular, a significantly high percentage of multi-drug failures was highlighted in patients with PsA and p-SpA, with a wider rate of tumor necrosis factor α inhibitors (TNFα-i) experienced (Table 4).

**Figure 1:**
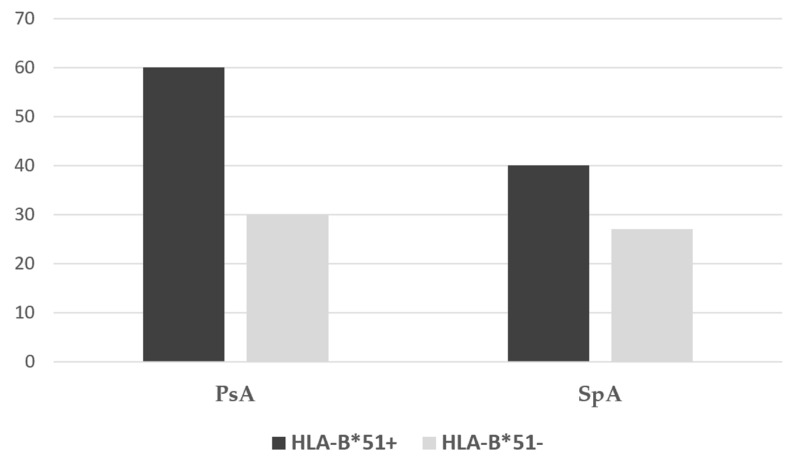
Rate of b-DMARDs line ≥3 in patients with PsA or SpA stratified for HLA-B*51 positivity or negativity.

Only a trend towards a higher frequency of multifailure with b-DMARDs was found in patients who were HLA-B*51 positive, compared to negative ones in both PsA and SpA (Figure 1). No differences were found in NSAID and DMARD user rates between HLA-B*51-positive and HLA-B*51-negative patients.

## 3. Discussion

In the era where growing scientific evidence agrees on the unification of AS, PsO, PsA, acute anterior uveitis, BD, and Birdshot’s disease into the unifying concept of MHC-I-pathy, it is important to publish a lot of real-life data on the particular impact that different classes of MHC-I class alleles can have on clinical manifestations. Some genetic factors are well-known predictors of disease susceptibility, such as HLA B*27 and AS, and HLA-A*29 and uveitis. Although the pathogenesis of BD is still unknown, an association between HLA-B*51 and BD has been observed, specifically in some ethnic groups from Turkey, Iran, and Japan [31,32,33].

For the first time, we presented data on the HLA-B*51 positivity in the Apulian population, who were clinically followed for more than 5 years, recording the clinical manifestations in order to monitor the achievement of the classification criteria for BD, SpA, and/or PsA.

Firstly, we found that 31% of patients met the BD classification criteria, supporting a discrete association between HLA-B*51 and BD, as previously described in the Italian population [20,22,34]

An interesting finding is the satisfaction of the classification criteria for BD and for SpA in 16% and 34% (ax-SpA and p-SpA, respectively) of patients. Although in the 1970s, Moll et al. proposed classifying BD within the seronegative spondyloarthropathies spectrum based on overlapping clinical features [35], this clinical concept remains arguable. Although axial involvement is rare in BD, increasing clinical data on the simultaneous overlap between BD and SpA are strengthening the concept of a possible clinical, genetic, and pathogenetic association [36,37,38,39]. In particular, the overlap between BD and SpA is frequently observed in HLA-B*27 and HLA-B*51 double-positive patients [27,39]. The high percentage of SpA among BD patients in our study, in the absence of HLA-B*27, could be due to a particular predisposition to SpA in the study population or to MRI, which allows for early diagnosis in patients with chronic inflammatory lower back pain.

Although the previously published data of a greater peripheral arthritis phenotype [25,26,27,28] is confirmed in our study as well, our BD patients with axial involvement presented a higher neurological involvement and a wider rate of IBD. These findings are not reported in other studies. Fascinating theories on microbial triggers and the key role of the gut microbiota in the pathogenic processes of BD could explain this latter association [40,41]. These hypotheses could be confirmed by the significant difference in the frequency of IBD between patients with HLA-B*51+ and those with negative HLA-B*51, supporting the likely importance of HLA-B*51 in its impact on these clinical phenotypes. Previous studies [21,22] on the possible association between HLA B*51 and more severe forms of BD could explain the higher percentage of neurological involvement in patients with BD and axial involvement, although no specific reference is made to these works.

Another interesting point is the higher prevalence of the female sex in our population of BD patients. Although BD was historically considered to be more prevalent in males, recent epidemiological data from Western and East Asian populations show a clear shift toward female predominance [42,43,44]. This is consistent with our findings and may be attributed to the higher frequency of mucocutaneous and articular manifestations in women.

For the first time, we tried to characterize the possible extra-articular manifestations in different phenotypes of arthritis, axial or peripheral, not only in BD, but also in SpA and PsA in patients who are HLA-B*51 positive. A significant percentage of patients with uveitis was found in SpA patients with predominant peripheral joint involvement. A previously published study on SpA and HLA-B*5 demonstrated a higher risk of the occurrence of oral ulcerations (and radiographic juxta-articular erosions) [27]. Only double positivity has been previously associated with a higher frequency of peripheral joint involvement, mucocutaneous manifestations (in particular oral aphthae), and uveitis [38]. But the absence of HLA-B*27 in our patients differentiates our study from the previously published one, opening new pathogenetic hypotheses. However, in our study, uveitis appears to be strictly associated with the HLA-B*51 positivity in the peripheral arthritis pattern, suggesting its probable pathogenetic importance in extra-articular manifestations in SpA.

There are no particular subsets of extra-articular manifestations that characterize the different phenotypes of axial and peripheral arthritis in PsA patients. A trend toward a higher recurrence of oral ulceration in patients with a peripheral pattern was observed without reaching statistical significance. Very few data are available on PsA and HLA-B*51 positivity [29]: they are predominantly in the Japanese population, with no particular clinical associations [45]. However, the finding of HLA-B*51 in 17 patients with PsA, as well as being an occasional finding, could lead to new clinical scenarios, and the small sample could justify the lack of characterization of extra-articular manifestations in the different joint phenotypes.

A speculative finding is the high number of multifailures to b-DMARDs (particularly TNFα-i) observed in HLA-B*51-positive patients with PsA and p-SpA. These results could be explained by a particular association between HLA-B*51 and the IL-17/23 axis. It has been observed that the simultaneous presence of HLA-B*51 and specific variants of the interleukin-23 receptor significantly increases susceptibility to the disease, with an immune polarization towards the Th17 phenotype. In this way, the majority of patients could benefit from inhibition of the IL-17/23 axis [46,47], although these results should be verified on a larger sample to validate them.

This study has some limitations: firstly, a small sample size, and secondly, as a single-center study on the Apulian population, it lacks verifiable external validity and ethnic variability in HLA-B*51 associations; but the exclusion of other genetic susceptibility variants enhances the validity of our data in a specific population. However, the primary objective of the study was to evaluate symptom development over a long observation period, which is useful for satisfying classification criteria for BD, SpA, and/or PsA in a patient population with a particular genetic susceptibility, such as HLA-B*51. Another limitation could be the time variable; it is known that symptoms can emerge slowly in both BD and SpA. Our long observation study time could compensate for this bias, but longer-term studies could be useful to strengthen these findings.

## 4. Materials and Methods

We conducted a monocentric retrospective study, evaluating the clinical records of a longitudinal cohort of 480 patients who were regularly admitted to our clinic and then evaluated for follow-up at our outpatient clinic from 2013 to 2025. The inclusion criteria were evaluation of HLA-B*51 positivity, the availability of the entire MHC-I class test, and rheumatological clinical follow-up of at least 5 years. The exclusion criterion was positivity for other clinically important MCH-I loci, such as principally HLA-B*15, B*27, B*57, HLA-A*26, HLA-C*14:02:01, C*06:02, and others, as indicated by the EULAR study group on ‘MHC-I-opathy’ [14].

Patients who did not meet any classification criteria during follow-up were defined as “Controls HLA-B*51+”.

To verify the clinical impact of HLA-B*51 positivity, patients with HLA-B*51 (as well as other clinically important MCH-I loci) negative status were selected using propensity score matching.

### 4.1. Materials and Methods

Demographic data (age, gender), clinical characteristics (anterior or posterior uveitis, retinal vasculitis), skin lesions (erythema nodosum-like, papulopustular lesions, pseudofolliculitis), vascular lesions, neurological manifestations, PsO, Pathergy test results (if available), joint imaging findings, joint involvement types (oligoarticular or polyarticular), presence of enthesitis, axial involvement (defined by the presence of inflammatory back pain and by radiographic evidence or magnetic resonance imaging of sacroiliitis or spondylitis), and the disease activity scores (DAS 28 [48] and ASDAS [49]) were collected at the baseline visit and at follow-up visits. The patients’ treatments were recorded at the baseline. All patients’ clinical data were collected in the electronic record used in our outpatient clinic, as is standard practice in our clinical practice.

The MHC-I class tests were performed at the referral genetic laboratory of our Policlinico and recorded in the electronic folders in use.

#### 4.1.1. Classification Criteria

In the study period, we evaluated whether and when the patients satisfied almost one of the following classification criteria: the Assessment of Spondyloarthritis International Society (ASAS) 2009 [50] or 2011 [51] criteria for ax-SpA or p-SpA, International Study Group (ISG) [52] and/or International Criteria for Behçet’s Disease (ICBD) [53] criteria for BD, and CASPAR criteria for PsA [54]. Diagnoses were revised based on the symptoms recorded for each patient over the follow-up period.

#### 4.1.2. Propensity Score Matching

To verify the real impact of HLA-B*51 on clinical manifestations, we selected a group of patients who were negative for HLA-B*51. To reduce the probable confounding factors, propensity score matching was used to select these patients who were HLA-B*51 negative, using age, gender, and disease as matching variables. The matching analysis was executed by the MatchIt package in R software (4.5.3 version, New Zealand), applying a nearest-neighbor algorithm with a 1:1 matching ratio without replacement.

#### 4.1.3. Statistical Analysis

The results were expressed as means (m) and standard deviations (SD); categorical variables were expressed as counts (percentages). The normality of the study variables’ distributions was assessed using the Kolmogorov–Smirnov test.

Statistical differences between the two groups were analyzed using Student’s *t*-test for unpaired data. Multiple comparisons of continuous variables were performed using analysis of variance (ANOVA), followed by the Bonferroni test.

Pearson χ2 and Fisher’s exact test, followed by the *Z*-test, were used to compare categorical variables and percentages.

The missing data ranged between 3% and 9%. Therefore, data imputation was not performed.

Statistical significance was defined as a value of *p* ≤ 0.05.

Statistical analysis was performed with IBM SPSS Statistics 26 (Armonk, NY, USA).

### 4.2. Local Ethical Committee Approval

This study was approved by the local ethics committee (Ethics Review Board of Policlinico of Foggia, protocol number 49/CE/2019), and all patients were informed about the nature and aim of the study and provided signed consent to participate.

## 5. Conclusions

This study provides new data on the association between HLA-B*51 and the onset of BD and/or SpA or PsA, and its possible impact on extra-articular manifestations. Furthermore, while it confirms the higher prevalence of the peripheral articular phenotype in BD, it also highlights a specific association between the rarer axial involvement and gastrointestinal involvement in HLA-B*51 patients. In SpA, the peripheral articular phenotype appears to be associated with a higher occurrence of uveitis in the presence of HLA-B*51. Further studies are essential to strengthen these findings and find new clinical associations, particularly in PsA patients. Multicenter studies would be desirable in order to verify the external validity and ethnic variability.

## Figures and Tables

**Table 1:** Demographic characteristics and laboratory findings at the baseline visit, classification criteria satisfied during the follow-up, extra-articular manifestation observed during the follow-up, and arthritis phenotypes in different patient groups with HLA B51+.

HLA-B*51+ Patients	BD n = 32	ax-SpA n = 13	p-SpA n = 25	PsA n = 17	Controls n = 18
Baseline Characteristics					
Age in years mean (SD)	42.6 (13.3)	32.5 (12.5)	44.2 (12.5)	51.9 (10.2)	44.6 (14.3)
Gender n (%)					
Males	11 (34)	6 (46)	6 (24)	8 (47)	4 (22)
Females	21 (66)	7 (54)	19 (76)	9 (53)	14 (78)
C-reactive protein mg/dL mean (SD)	1.5 (0.5)	2.5 (1.3)	2.1 (0.9)	1.9 (1.8)	-
Arthritis phenotypes					
Oligo-arthritis n (%)	1 (3)	0 (0)	0 (0)	0 (0)	-
Poly-arthritis n (%)	17 (53)	8 (61)	25 (100)	15 (88)	-
Enthesitis n (%)	8 (25)	6 (46)	21 (84)	13 (76)	-
Sacroiliitis n (%)	5 (16)	13 (100)	0 (0)	7 (41)	-
Syndesmophytes n (%)	5 (16)	13 (100)	2 (8)	6 (35)	-
Joint disease activity at baseline					
ASDAS score mean (SD)	1.8 (0.7)	2.6 (1.2)	1.9 (0.9)	2.5 (1.7)	-
DAS 28 CRP mean (SD)	2.2 (1.8)	1.7 (2.3)	2.7 (1.4)	3.2 (1.5)	-
**Characteristics observed in follow-up**					
ISG criteria n (%)	26 (81)	0 (0)	0 (0)	0 (0)	0 (0)
ICBD criteria n (%)	27 (84)	0 (0)	0 (0)	0 (0)	0 (0)
ax-SpA ASAS criteria n (%)	5 (16)	13 (100)	-	-	0 (0)
p-SpA ASAS criteria n (%)	11 (34)	-	25 (100)	-	0 (0)
CASPAR criteria n (%)	0 (0)	-	-	17 (100)	0 (0)
Diagnostic delay mean (SD)	8.4 (2.9)	8.0 (2.3)	7.2 (2.0)	6.5 (1.8)	-
Extra-articular manifestations					
Psoriasis n (%)	2 (6)	0 (0)	2 (8)	11 (65)	0 (0)
Nail psoriasis n (%)	0 (0)	0 (0)	0 (0)	8 (49)	0 (0)
Cutaneous lesions n (%)	16 (50)	1 (8)	6 (24)	6 (35)	3 (17)
Anterior uveitis n (%)	4 (12)	0 (0)	1 (17)	1 (6)	0 (0)
Posterior uveitis n (%)	8 (25)	0 (0)	6 (24)	1 (6)	3 (17)
Neurological lesion n (%)	4 (12)	0 (0)	1 (4)	0 (0)	1 (6)
Recurrent oral ulcerations n (%)	31 (97)	4 (38)	4 (16)	4 (23)	2 (11)
Recurrent genital ulceration n (%)	20 (62)	0 (0)	0 (0)	0 (0)	1 (6)
Vascular lesions n (%)	8 (25)	1 (8)	2 (8)	0 (0)	0 (0)
Inflammatory bowel disease n (%)	9 (28)	3 (23)	5 (20)	2 (12)	2 (11)
Serositis n (%)	2 (6)	0 (0)	4 (16)	1 (6)	0(0)

**Table 2:** Comparison of demographic and clinical characteristics between HLA-B*51-positive and HLA-B*51-negative patients.

	HLA-B*51+ n = 87	HLA-B*51- n = 87	*p*-Value
Baseline Characteristics			
Age	44.7 (10.6)	44.5 (11.6)	0.919
Gender n (%)			
Males	31 (36)	31 (36)	**-**
Females	56 (64)	56 (64)	-
Arthritis phenotypes			
Oligo-arthritis n (%)	1 (1)	0 (0)	0.978
Poly-arthritis n (%)	64 (74)	72 (83)	0.247
Enthesitis n (%)	48 (55)	49 (56)	0.879
Sacroiliitis n (%)	20 (23)	24 (28)	0.485
Syndesmophytes n (%)	26 (30)	12 (13)	0.010
**Characteristics observed in follow-up**			
Extra-articular manifestations			
Psoriasis n (%)	15 (17)	18 (21)	0.139
Cutaneous lesions n (%)	29 (33)	18 (21)	0.139
Uveitis n (%)	21 (24)	14 (16)	0.186
Neurological lesion n (%)	5 (38)	8 (9)	0.387
Recurrent oral ulceration (%)	43 (49)	36 (41)	0.286
Recurrent genital ulceration n (%)	20 (23)	27 (31)	0.232
Vascular lesions n (%)	11 (13)	6 (7)	0.202
IBD n (%)	19 (22)	18 (21)	0.853
Serositis n (%)	7 (8)	3 (3)	0.193
BD diagnosis	32 (37)	32 (37)	-
ISG criteria n (%)	26/32 (81)	21/32 (66)	-
ICBD criteria n (%)	27/32 (84)	30/32 (93)	-
ax-SpA ASAS criteria n (%)	13 (15)	13 (15)	-
p-SpA ASAS criteria n (%)	25 (29)	25 (29)	-
CASPAR criteria	17 (19)	17 (19)	-

**Table 3:** Comparison of extra-articular manifestations between peripheral and axial involvement in Behcet’s disease (BD), spondyloarthritis (SpA), and psoriatic arthritis (PsA).

	HLA-B*51+		HLA-B*51-		*p*-Value			
	Axial Involvement (a)	Peripheral Arthritis (b)	Axial Involvement (c)	Peripheral Arthritis (d)	a vs. b	a vs. c	b vs. d	c vs. d
Behcet’s disease	n = 5	n = 27	n = 3	n = 29				
Psoriasis n (%)	1 (20)	1 (4)	2 (67)	2 (7)	0.307	0.187	0.166	0.03
Cutaneous lesions n (%)	3 (60)	13 (48)	0 (0)	17 (59)	0.626	0.090	0.432	0.053
Uveitis n (%)	1 (20)	17 (41)	2 (67)	9 (31)	0.379	0.187	0.449	0.216
Neurological lesion n (%)	2 (40)	2 (7)	2 (67)	6 (21)	0.043	0.465	0.156	0.080
Recurrent OU n (%)	5 (100)	26 (96)	3 (100)	27 (93)	0.662	-	0.596	0.638
Recurrent GU n (%)	3 (60)	17 (63)	3 (100)	24 (83)	0.634	0.206	0.095	0.434
Vascular lesions n (%)	0 (0)	8 (30)	0 (0)	6 (21)	0.160	-	0.440	0.382
IBD n (%)	5 (100)	4 (15)	1 (33)	6 (21)	0.0001	0.035	0.566	0.614
Serositis n (%)	0 (0)	2 (7)	1 (33)	1 (3)	0.734	-	0.476	0.042
SpA	n = 13	n = 25	n = 13	n = 25				
Psoriasis n (%)	0 (0)	2 (8)	0 (0)	0 (0)	0.429	-	0.245	-
Cutaneous lesions n (%)	1 (8)	6 (24)	0 (0)	1 (4)	0.219	0.172	0.063	0.465
Uveitis n (%)	0 (0)	7 (28)	0 (0)	1 (4)	0.035	0.343	0.030	0.465
Neurological lesion n (%)	0 (0)	1 (4)	0 (0)	0 (0)	0.465	-	0.292	-
Recurrent OU n (%)	4 (38)	4 (16)	1 (8)	1 (4)	0.289	0.191	0.129	0.629
Recurrent GU n (%)	0 (0)	0 (0)	0 (0)	0 (0)	-	-	-	-
Vascular lesions n (%)	1 (8)	2 (8)	0 (0)	0 (0)	0.973	0.343	0.132	-
IBD n (%)	3 (23)	5 (20)	6 (46)	4 (16)	0.825	0.139	0.611	0.045
Serositis n (%)	0 (0)	4 (16)	0 (0)	0 (0)	0.311	0.162	0.364	-
PsA	n = 7	n = 10	n = 10	n = 7				
Psoriasis n (%)	4 (57)	7 (70)	7 (70)	7 (100)	0.862	0.862	0.279	0.279
Cutaneous lesions n (%)	2 (29)	4 (40)	0 (0)	(0)	0.627	0.072	0.056	-
Uveitis n (%)	0 (0)	2 (11)	1 (10)	1 (14)	0.208	0.388	0.761	0.787
Neurological lesion n (%)	0 (0)	0 (0)	0 (0)	0 (0)	-	-	-	-
Recurrent OU n (%)	1 (14)	3 (30)	1 (10)	3 (43)	0.452	0.787	0.585	0.116
Recurrent GU n (%)	0 (0)	0 (0)	0 (0)	0 (0)	-	-	-	-
Vascular lesions n (%)	0 (0)	0 (0)	0 (0)	0 (0)	-	-	-	-
IBD n (%)	1 (14)	1 (10)	0 (0)	1 (14)	0.787	0.218	0.787	0.218
Serositis n (%)	0 (0)	1 (6)	0 (0)	1 (14)	0.343	-	0.218	0.218

GU: genital ulcerations; IBD: inflammatory bowel disease; OU: oral ulcerations; PsA: psoriatic arthritis.

**Table 4:** Treatment characteristics in Behcet’s disease (BD), axial spondyloarthritis (ax-SpA), peripheral spondyloarthritis (p-SpA), and psoriatic arthritis (PsA).

	BD n = 32	ax-SpA n = 13	p-SpA n = 25	PsA n = 17	*p*-Value
NSAID response n (%)	22 (70)	11 (85)	19 (78)	13 (80)	0.675
b-DMARDs n (%)	23 (72)	13 (100)	16 (64)	12 (70)	0.723
b-DMARDs Line ≥3	2/23 (9)	0 (0)	6/16 (40)	7/12 (60)	0.001 *
TNFα-i treatment, ever	17/23 (74)	13/13 (100)	12/16 (75)	10/12 (83)	0.456
IL17-i/IL12/23-i/IL23-i treatment ever	-	3/13 (23)	7/16 (44)	8/12 (67)	0.293

* *p* ≤ 0.05 PsA vs. ax-SpA; p-SpA vs. ax-SpA; PsA vs. BD; and p-SpA vs. BD. IL17-i: Interleukin 17 inhibitors, IL 12/23-i: interleukin 12/23 inhibitors; IL23-i: interleukin 23 inhibitors.

## Data Availability

The original contributions presented in this study are included in the article. Further inquiries can be directed to the corresponding author.

## Abbreviations

The following abbreviations are used in this manuscript:

AS	Ankylosing Spondylitis
ASAS	Assessment of Spondyloarthritis International Society
ASDAS	Ankylosing Spondylitis Disease Activity Score
ax-SpA	Axial Spondyloarthritis
b-DMARDs	Biologic Disease-Modifying Antirheumatic Drugs
BD	Behcet’s Disease
CRP	C-reactive Protein
DAS	Disease Activity Score
HLA	Human Leukocyte Antigen
GU	Genital Ulceration
IBD	Inflammatory Bowel Disease
ICBD	International Criteria for Behçet’s Disease
IL	Interleukine
ISG	International Study Group
MHC-I	Major Histocompatibility Complex class I
NSAID	Nonsteroidal Anti-Inflammatory Drug
OU	Oral Ulceration
PsA	Psoriatic Arthritis
p-SpA	Peripheral Spondyloarthropathy
TNFα-i	Tumor Necrosis Factor A Inhibitors
